# Mouse Type-I Interferon-Mannosylated Albumin Fusion Protein for the Treatment of Chronic Hepatitis

**DOI:** 10.3390/ph17020260

**Published:** 2024-02-19

**Authors:** Yuki Minayoshi, Hitoshi Maeda, Keisuke Hamasaki, Taisei Nagasaki, Mei Takano, Ryo Fukuda, Yuki Mizuta, Motohiko Tanaka, Yutaka Sasaki, Masaki Otagiri, Hiroshi Watanabe, Toru Maruyama

**Affiliations:** 1 Department of Biopharmaceutics, Graduate School of Pharmaceutical Sciences, Kumamoto University, 5-1 Oe-Honmachi, Chuo-ku, Kumamoto 862-0973, Japan; 233y2007@st.kumamoto-u.ac.jp (Y.M.); 217y2002@st.kumamoto-u.ac.jp (K.H.); t.nagasaki0507@gmail.com (T.N.); 221y3004@st.kumamoto-u.ac.jp (M.T.); 220y3005@st.kumamoto-u.ac.jp (R.F.); 222y2006@st.kumamoto-u.ac.jp (Y.M.); hnabe@kumamoto-u.ac.jp (H.W.); 2 Department of Gastroenterology and Hepatology, Graduate School of Medical Sciences, Kumamoto University, 1-1-1 Honjo, Chuo-ku, Kumamoto 860-8556, Japan; mtanaka03@kumamoto-u.ac.jp (M.T.); sasakiy@kumamoto-u.ac.jp (Y.S.); 3 Public Health and Welfare Bureau, 5-1-1 Oe, Chuo-ku, Kumamoto 862-0971, Japan; 4 Osaka Central Hospital, 3-3-30 Umeda, Kita-ku, Osaka 530-0001, Japan; 5 Faculty of Pharmaceutical Sciences, Sojo University, 4-22-1 Ikeda, Nishi-ku, Kumamoto 860-0082, Japan; otagirim@ph.sojo-u.ac.jp; 6 DDS Research Institute, Sojo University, 4-22-1 Ikeda, Nishi-ku, Kumamoto 860-0082, Japan

**Keywords:** type-I interferon, mannose receptor, Kupffer cells, albumin fusion technology, site-specific mutagenesis, species difference

## Abstract

Although a lot of effort has been put into creating drugs and combination therapies against chronic hepatitis, no effective treatment has been established. Type-I interferon is a promising therapeutic for chronic hepatitis due to its excellent anti-inflammatory effects through interferon receptors on hepatic macrophages. To develop a type-I IFN equipped with the ability to target hepatic macrophages through the macrophage mannose receptor, the present study designed a mouse type-I interferon-mannosylated albumin fusion protein using site-specific mutagenesis and albumin fusion technology. This fusion protein exhibited the induction of anti-inflammatory molecules, such as IL-10, IL-1Ra, and PD-1, in RAW264.7 cells, or hepatoprotective effects on carbon tetrachloride-induced chronic hepatitis mice. As expected, such biological and hepatoprotective actions were significantly superior to those of human fusion proteins. Furthermore, the repeated administration of mouse fusion protein to carbon tetrachloride-induced chronic hepatitis mice clearly suppressed the area of liver fibrosis and hepatic hydroxyproline contents, not only with a reduction in the levels of inflammatory cytokine (TNF-α) and fibrosis-related genes (TGF-β, Fibronectin, Snail, and Collagen 1α2), but also with a shift in the hepatic macrophage phenotype from inflammatory to anti-inflammatory. Therefore, type-I interferon-mannosylated albumin fusion protein has the potential as a new therapeutic agent for chronic hepatitis.

## 1. Introduction

Chronic hepatitis is caused by a variety of factors, including viral infection [1], alcohol consumption [2], lifestyle [3], and autoimmunity [4], all of which lead to liver fibrosis in more advanced stages [5]. In the initial stage of chronic hepatitis, hepatic macrophages (Kupffer cells) are activated by both endogenous ligands generated from injured cells and exogenous ligands generated from the gut microbiota [6]. The activated Kupffer cells produce inflammatory cytokines, leading to the destruction of hepatocytes and the activation of the hepatic stellate cells (HSCs) [6,7]. The activated HSCs start to produce an extracellular matrix, including collagen, to repair the architecture of the injured liver, in a manner similar to the process of wound healing [8]. As a result of the aforementioned processes being repeated, fibrosis eventually progresses from localized to entire lobes of the liver. Therefore, inhibiting the activation of Kupffer cells and HSCs is the key to alleviating the severity of chronic hepatitis.

Since interferon (IFN) was discovered as a “virus suppressor” in 1953 [9], a variety of biological activities have been discovered, such as an anti-tumor effect [10], a proliferation-inhibiting effect [11], and a cell differentiation effect [12]. It has also recently been shown that type-I IFN (IFN-α and β) has the ability to induce IL-10 (an anti-inflammatory cytokine) [13], IL-1 receptor antagonist (IL-1Ra, an inflammasome inhibitor) [14], and programmed death-1 (PD-L1, an immune checkpoint molecule) [15,16] when it acts on the IFN receptors of macrophages. These findings show that type-I IFN is a promising therapeutic molecule for inflammatory and autoimmune diseases.

Contrary to expectations, type-I IFN is also known to aggravate immune-related disorders [17,18]. In fact, there have been several cases where patients who received IFN-α developed autoimmune hepatitis [19]. The anti-inflammatory effects of type-I IFN have been understood to depend on its amount of accumulation on immune cells [17]. In other words, type-I IFN exacerbates autoimmune reactions if it acts on macrophages at low concentrations [17]. These findings prompted us to hypothesize that type-I IFN induces IL-10, IL-1Ra, and PD-L1 by selectively delivering type-I IFN to macrophages. Therefore, we tried to develop a type-I IFN that accumulates on macrophages at high concentrations.

However, two pharmacokinetic challenges stood in the way of developing such a type-I IFN. The first is that type-I IFN is not equipped with the ability to target macrophages. Since the proportion of macrophages present in organs is overwhelmingly lower than that of parenchymal cells, there is a concern that the administered type-I IFN would be mainly distributed to parenchymal cells. The second is the limited hepatic bioavailability of type-I IFN. Since the molecular weight of type-I IFN is 19 kDa [20], administered type-I IFN would mostly be excreted from the body through glomerular filtration. Therefore, to overcome these issues, it is essential to develop a drug delivery system (DDS) that can selectively deliver type-I IFN to Kupffer cells while avoiding glomerular filtration.

Since human serum albumin (HSA) is a simple protein with a molecular weight of approximately 66 kDa, HSA is rarely eliminated due to glomerular [21]. HSA has been widely used as a DDS carrier because of its high biocompatibility and biodegradability, as well as its excellent drug-loading ability [22]. There are many applications of HSA that aim to improve the retention rate of functional proteins in blood [23]. In particular, albumin fusion technology, which fuses albumin with small functional proteins or peptides at the genetic level, has been established as one of the platforms for albumin DDS [24]. To date, using this technology, more than 40 instances of chimeric proteins have been designed [25]. Alternatively, we previously developed a high-mannosylated albumin that targets the mannose receptor expressed on Kupffer cells [26] and demonstrated its usefulness as a carrier for targeting Kupffer cells [27,28,29]. Specifically, we inserted *N*-glycosylation sequences into wild-type albumin using site-specific mutagenesis and expressed this mutated protein in *Pichia pastoris*. The resulting albumin contains bulky sugar chains (11 mannose residues) that are characteristic of the *Pichia pastoris* expression system. In fact, based on this unique sugar chain structure, high-mannosylated albumin is preferentially recognized by Kupffer cells, and, as a result, is rapidly eliminated from the blood and efficiently distributed to the liver. Therefore, by combining this high-mannosylated albumin with the albumin fusion technology described above, it is theoretically possible to develop a type-I IFN equipped with the ability to target Kupffer cells.

In the present study, based on this background information, we attempted to develop a mouse type-I IFN chimeric protein, because it is known that there is a species difference in IFN activity [30]. Albumin fusion technology was used to fuse high-mannosylated albumin, a Kupffer cell-targeted carrier, with type-I IFN, thereby avoiding glomerular filtration and improving the hepatic bioavailability. We expressed the chimeric fusion protein in *Pichia pastoris* and analyzed the biological activity of the protein in the macrophage cell line. Furthermore, the effectiveness of the chimeric fusion protein on chronic hepatitis was verified by the repeated administration of this chimeric fusion to a carbon tetrachloride (CCl_4_)-induced chronic hepatitis model in mice.

## 2. Results

### 2.1. Production and Biological Activity of Man-MSA-mIFNα2

**Figure 1:**
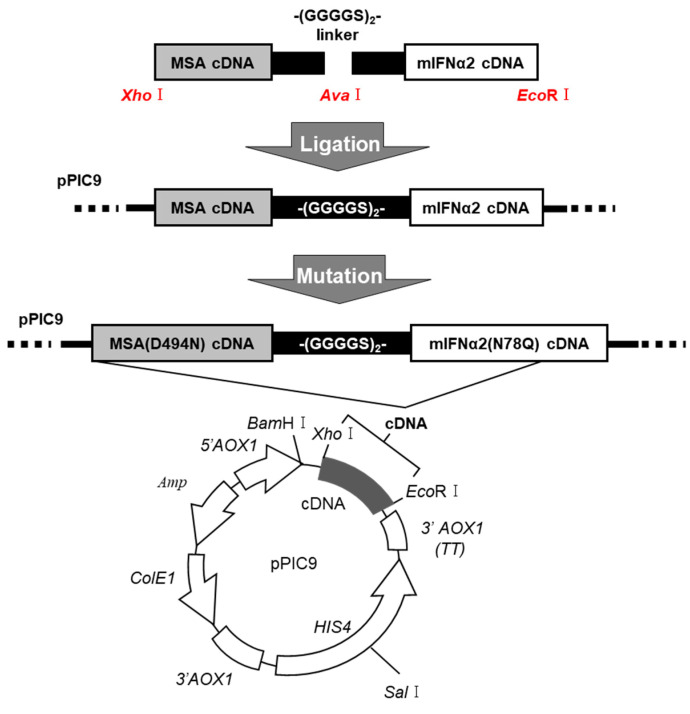
Flow chart describing the creation of the Man-MSA(D494N)-mIFNα2(N78Q) gene using the pPIC9.

We previously developed a fusion of human IFNα2b with mannosylated human serum albumin (Man-HSA-hIFNα2b) [31]. The present study designed a new fusion of mouse IFNα2 with mannosylated mouse serum albumin (Man-MSA-mIFNα2) to improve the biological activity of type-I IFN. A fusion of MSA and mIFNα2 was developed according to our previous method. As shown in Figure 1, MSA and mIFNα2 cDNAs were ligated via a peptide linker (-(Gly-Gly-Gly-Gly-Ser)_2_-), then the cDNA was inserted into the cloning sites (EcoR1 and Xho1) on the pPIC9 vector, which includes multiple cloning sites and is often used to express any proteins in *Pichia pastoris*. To introduce a glycosylation sequence into MSA, aspartic acid at position 494 of MSA was replaced with asparagine (D494N). Furthermore, to remove a glycosylation sequence originally present in mIFNα2, asparagine at position 78 of mIFNα2 was replaced with glutamic acid (N78Q). After the introduction of the prepared pPIC9-MSA(D494N)-mIFNα2(N78Q) into *Pichia pastoris*, fusion proteins were expressed in a culture of the *Pichia pastoris* using methanol as a carbon source, and then purified using a conventional method [31].

**Figure 2:**
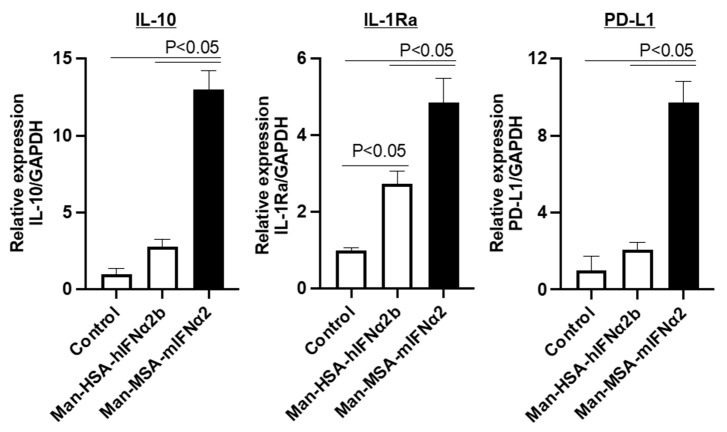
Anti-inflammatory and immunomodulatory effects of Man-MSA-mIFNα2 on RAW264.7 cells. mRNA expression levels of IL-10, IL-1Ra, and PD-L1 were evaluated 3 h after treatment of RAW264.7 cells with Man-HSA-hIFNα2b or Man-MSA-mIFNα2.

To confirm the biological activity of Man-MSA-mIFNα2, the mRNA expression levels of IL-10, IL-1Ra, and PD-L1 were quantified 3 h after treatment of RAW264.7 cells, murine macrophage-like cells, with Man-MSA-mIFNα2 or Man-HSA-hIFNα2b (Figure 2). As a result, the induction activities of IL-10, IL-1Ra, and PD-L1 by Man-MSA-mIFNα2 were approximately 5 times, 2 times, and 4.5 times higher, respectively, than Man-HSA-hIFNα2b.

### 2.2. CCl_4_-Induced Chronic Hepatitis Model Mouse

**Figure 3:**
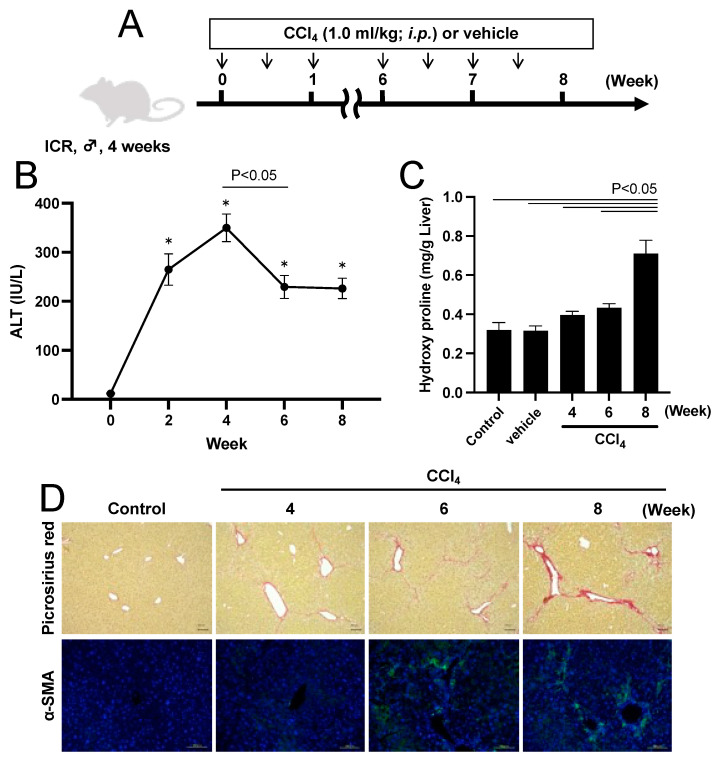
Pathological phenotypes in CCl_4_-induced hepatitis mice. (**A**) Schematic summary of the experimental protocol for preparing CCl_4_-induced hepatitis mice. (**B**) Plasma ALT values were determined at 0, 2, 4, 6, and 8 weeks after repeated administration of CCl_4_ (1.0 mL/kg, *i.p.*). Each value represents the mean ± S.E. (n = 5). * *p* < 0.05 compared with week 0. (**C**) The levels of hepatic hydroxyproline were determined at 0, 4, 6, and 8 weeks after repeated administration of CCl_4_. Each value represents the mean ± S.E. (n = 5). (**D**) Liver fibrosis was evaluated by picrosirius red staining and immunofluorescence staining of α-SMA (green) with DAPI (blue). Scale bars, 100 µm.

CCl_4_ is widely used as a pathological model and for mechanistic research into liver fibrosis [32]. Hepatic cytochrome P450 metabolized CCl_4_ into highly reactive free radicals (•CCl_3_) which induce lipid peroxidation, resulting in hepatocellular damage. Damage-associated molecular patterns derived from necrotic hepatocytes activate Kupffer cells. The sustained activation of Kupffer cells by the repeated administration of CCl_4_ further activates HSCs, resulting in the progression of liver fibrosis. Therefore, we prepared a CCl_4_-induced chronic hepatitis model by intraperitoneally administering CCl_4_ (1.0 mL/kg) to ICR mice twice a week, according to our previous report [33] (Figure 3A).

To determine the timing to start the administration of Man-MSA-mIFNα2, we evaluated the pathological phenotypes in this model over a period of time. In chronic hepatitis, the number of hepatocytes is known to decrease due to hepatocellular damage, resulting in a gradual decrease in alanine aminotransferase (ALT) values during the progression of pathological conditions. Therefore, we first measured serum ALT values at 0, 2, 4, 6, and 8 weeks after starting CCl_4_ administration, and observed an increase in ALT values from 0 to 4 weeks, and a decrease at 6 to 8 weeks (Figure 3B). To evaluate collagen accumulation in the liver, we next quantified hepatic hydroxyproline contents, the main component of collagen, and found that it increased slightly from week 4, and was significantly increased at week 8 (Figure 3C). To observe collagen fibers, we also performed picrosirius red staining, and observed fibrous hepatic tissue around the sinusoids connecting the portal vein and central vein from week 4, and found remarkable aggravation of this finding at week 8 (Figure 3D, upper panel). Activated HSCs by inflammatory cytokines and growth factors produce collagen. Thus, we confirmed the expression of α-SMA, which is an indicator of the activation for HSCs, by immunofluorescence staining and observed α-SMA positive areas from week 4 (Figure 3D, lower panel).

### 2.3. Effect of Man-MSA-mIFNα2 on Hepatocellular Damage in CCl_4_-Induced Chronic Hepatitis Mice

Since our results showed that liver fibrosis in a CCl_4_-induced chronic hepatitis model progressed rapidly from 6 to 8 weeks after twice weekly administrations of CCl_4_, we set the timing of therapeutic intervention of Man-MSA-mIFNα2 from week 6 after starting the repeated administration of CCl_4_. To also confirm the biological activity of mouse type-I IFN in vivo, we first administered Man-MSA-mIFNα2 or Man-HSA-hIFNα2b to CCl_4_-induced chronic hepatitis mice (Supplemental Figure S1A) and compared serum ALT values. We administered each fusion of type-I IFN with mannosylated albumin to the mice 24 h after CCl_4_ treatment, because the lipid peroxidation induced by CCl_4_ was assumed to have been sufficiently completed at that timing according to the previous report [34]. ALT values showed large variations within the Man-HSA-hIFNα2b-administred group, but the values tended to decrease in the Man-MSA-mIFNα2-administred group (Supplemental Figure S1B). This result shows that mIFNα2 with mouse mannosylated albumin exhibited a superior hepatoprotective effect on chronic hepatitis mice than that of the human fusion protein. As a result, the therapeutic effects of Man-MSA-mIFNα2 on chronic hepatitis were evaluated in more detail in subsequent analyses.

**Figure 4:**
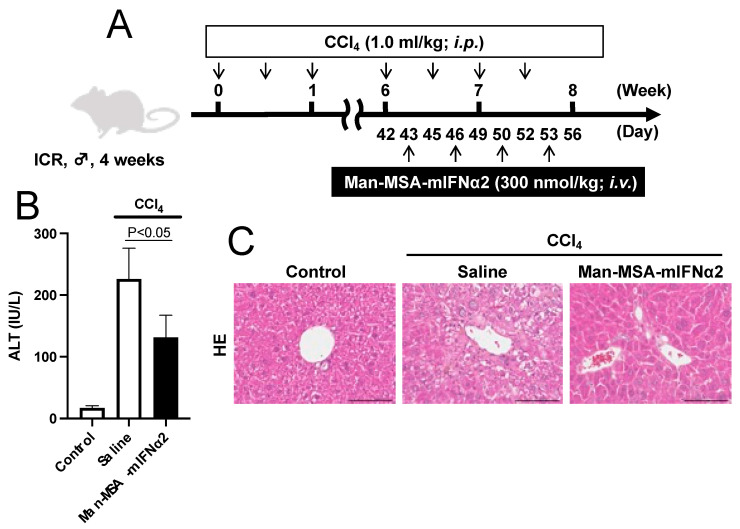
Effect of Man-MSA-mIFNα2 on hepatocellular damage in CCl_4_-induced chronic hepatitis mice. (**A**) Schematic summary of the experimental protocol for evaluation of the effect of Man-MSA-mIFNα2 on hepatocellular damage on CCl_4_-induced chronic hepatitis mice. (**B**) Plasma ALT values were determined 8 weeks after repeated administration of CCl_4_ (1.0 mL/kg, *i.p.*). Each value represents the mean ± S.E. (n = 5). (**C**) Sections of liver tissue were prepared 8 weeks after repeated administration of CCl_4_ and subjected to histopathological examination (HE staining). Scale bars, 100 µm.

According to the schedule shown in Figure 4A, we intravenously administered Man-MSA-mIFNα2 to CCl_4_-induced chronic hepatitis mice and investigated the effect on hepatocellular damage 8 weeks after repeated administration of CCl_4_. As shown in Figure 4B, increased plasma ALT values by repeated administration of CCl_4_ were significantly reduced by Man-MSA-mIFNα2 administration. We also performed HE staining as a histopathological evaluation of the liver, and found that nuclear dropout and necrosis, which were occasionally observed in the saline-treated group, were mitigated in the Man-MSA-mIFNα2-administred group (Figure 4C).

### 2.4. Hepatoprotective Mechanism of Man-MSA-mIFNα2 against CCl_4_-Induced Chronic Hepatitis Mice

**Figure 5:**
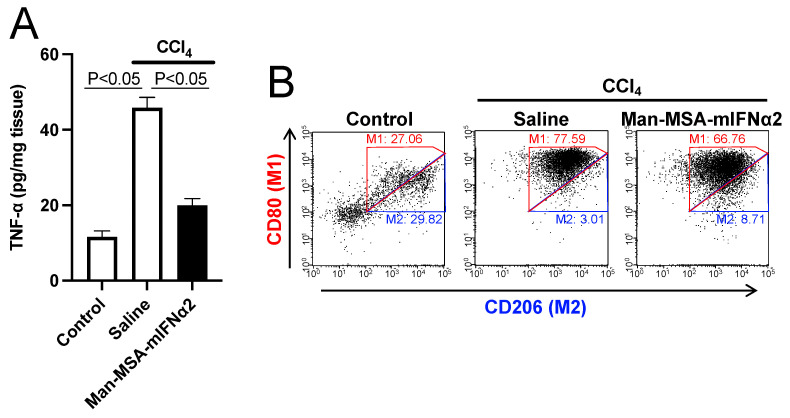
Effects of Man-MSA-mIFNα2 on inflammatory cytokines or macrophage polarization in CCl_4_-induced chronic hepatitis mice. (**A**) The expression levels of TNF-α in liver were determined by ELISA. Each value represents the mean ± S.E. (n = 5). (**B**) Hepatic macrophages were isolated from the livers of CCl_4_-induced chronic hepatitis mice, and the populations of M1 or M2 macrophages were analyzed using CD80 (M1 marker) or CD206 (M2 marker), respectively.

Since inflammatory cytokines, such as tumor necrosis factor-α (TNF-α) derived from Kupffer cells, play an important role in the progression of CCl_4_-induced hepatitis [35], the effect of Man-MSA-mIFNα2 on TNF-α production was investigated using ELISA (Figure 5A). A marked increase in hepatic TNF-α was observed in the saline-treated group. However, this increase was significantly suppressed in the Man-MSA-mIFNα2-administred group. This result shows that the suppression of inflammatory cytokines by Man-MSA-mIFNα2 administration contributed to the alleviation of hepatocellular damage in CCl_4_-induced chronic hepatitis mice.

Since macrophages are classified into inflammatory macrophages (M1) and anti-inflammatory macrophages (M2), we isolated hepatic macrophages from the livers of CCl_4_-induced chronic hepatitis mice and analyzed the populations of positive cells for CD80 (M1 marker) or CD206 (M2 marker) by flow cytometry (Figure 5B) according to the previous report [36]. As a result, Man-MSA-mIFNα2 administration decreased the populations of M1 cells up to approximately 67% and, conversely, increased the populations of M2 cells by approximately 9%. We also found that Man-MSA-mIFNα2 decreased the ratio of M1 and M2 (M1/M2) from 25.78 to 7.66, indicating that the macrophage phenotype may have shifted from inflammatory to anti-inflammatory (Table 1).

### 2.5. Effect of Man-MSA-mIFNα2 on Liver Fibrosis in CCl_4_-Induced Chronic Hepatitis Mice

**Figure 6:**
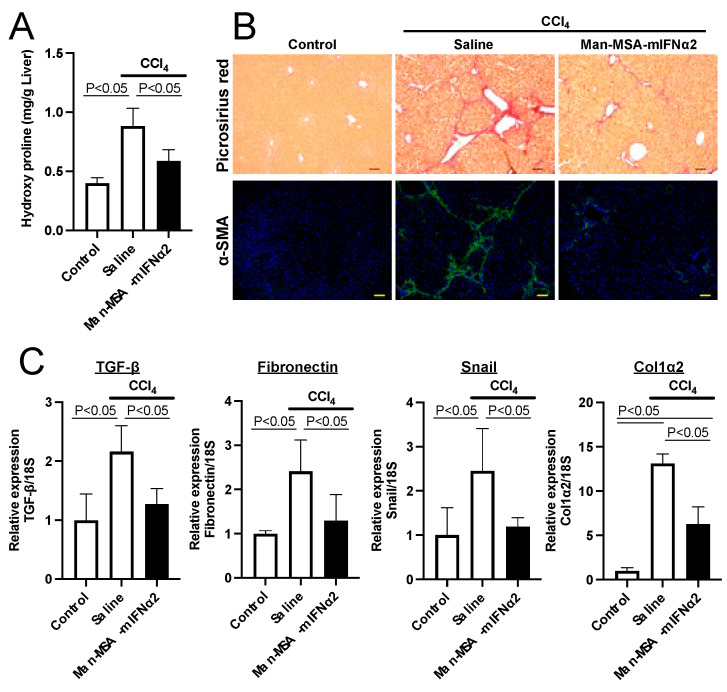
Effect of Man-MSA-mIFNα2 on liver fibrosis in CCl_4_-induced chronic hepatitis mice. (**A**) Hepatic hydroxyproline contents were determined 8 weeks after repeated administration of CCl_4_. Each value represents the mean ± S.E. (n = 5). (**B**) Liver fibrosis was evaluated by picrosirius red staining and immunofluorescence staining of α-SMA (green) with DAPI (blue). Scale bars, 100 µm. (**C**) The mRNA expression levels of TGF-β, Fibronectin, Snail, and Collagen 1α2 in liver were determined by qRT-PCR. Each value represents the mean ± S.E. (n = 5).

We quantified hepatic hydroxyproline contents in CCl_4_-induced chronic hepatitis mice and found that the elevated hydroxyproline contents due to the repeated administration of CCl_4_ were significantly reduced by Man-MSA-mIFNα2 administration (Figure 6A). Similar results were also obtained from picrosirius red staining, in which collagen fibers were observed from the central vein to the portal vein in the saline-administered group, whereas there was obvious suppression of fibrosis progression in the Man-MSA-mIFNα2 administration group (Figure 6B upper panel). We also confirmed the expression of α-SMA using immunofluorescence staining and observed that the increased α-SMA-positive area in the saline-administered group was obviously reduced in the Man-MSA-mIFNα2-administred group (Figure 6B lower panel). We further investigated the effects of Man-MSA-mIFNα2 on the mRNA expression levels of TGF-β, Fibronectin, Snail, and Collagen 1α2, which are factors that promote fibrosis (Figure 6C). As a result, the elevated levels of these factors found in the saline-administered group were shown to be significantly suppressed in the Man-MSA-mIFNα2-administered group. These results show that Man-MSA-mIFNα2 mitigates the progression of liver fibrosis.

## 3. Discussion

Based on the results of the large-scale clinical trial conducted by Angulo et al., it was found that liver fibrosis is an important factor in determining the long-term prognosis of non-alcoholic steatohepatitis (NASH) [37]. Therefore, a lot of effort has been invested in creating drugs and combination therapies, which have not only “hepatoprotective effects” but also “antifibrotic effects” on chronic hepatitis, including NASH [38]. However, at the time of writing, no effective treatment for chronic hepatitis has been established.

Type I IFNs have been shown to be effective against various diseases, such as polyethylene glycol (PEG)-modified IFN-α2b (PegIntron) [39] and next-generation pegylated interferon (Ropeginterferon α2b) [40,41] for HCV and HBV, IFN-α (Sumiferon) for renal cancer and multiple myeloma [42], and IFN-β1b (Betaseron) and IFN-β1a (Avonex) for multiple sclerosis [43], but their underlying mechanisms of action are different. For example, when type I IFN acts on IFN receptors expressed on infected cells, i.e., hepatic parenchymal cells, it inhibits virus proliferation by inducing (2′–5′) oligoadenylate synthase protein kinase and antiviral proteins (Mx), thereby exerting antiviral effects. On the other hand, when type I IFN directly acts on cancer cells, it suppresses proliferation by controlling the cell cycle, in addition to the activation of immune cells such as NKT and CD8+ T cells, resulting in an antitumor effect. Furthermore, when type I IFN acts on the peripheral and central T cells, it inhibits their activation, in addition to the suppression of autoimmune reactions by decreasing the expression of MHC class II molecules on macrophages, thus controlling the pathological conditions of multiple sclerosis.

Interestingly, type-I IFN also shows organ-protective [44] and antifibrotic actions [45,46,47,48,49,50,51] through the interaction with its receptors on macrophages. These findings led us to the idea that type-I IFN, equipped with the ability to target Kupffer cells, has potential as a therapeutic for chronic hepatitis. To achieve this, the present study solved two pharmacokinetic limitations of type-I IFN—the lack of ability to target macrophages and the limited hepatic bioavailability due to glomerular filtration—by combining albumin site-specific mutagenesis and albumin fusion technology (Figure 1). Specifically, we created a fusion of albumin with high-mannose chains and type-I IFN through the design of mannosylated albumin using site-specific mutagenesis, the genetical fusion of mannosylated albumin and type-I IFN, and the expression of the fusion protein in *Pichia pastoris*. This fusion protein exerted excellent hepatoprotective and antifibrotic effects in chronic hepatitis model mice by improving the bioavailability of type-I IFN in Kupffer cells as a result of the high-mannose chains of mannosylated albumin being recognized by the macrophage mannose receptor.

As shown in Figure 1, the present study selected aspartic acid at position 494 of albumin as the site for introducing mannose chains. Approximately 100 variants of HSA have been found [52,53,54,55], one of which is the D494N mutation. This variant was discovered in a family residing in Casebrook, New Zealand, and is therefore called “Albumin Casebrook” [54]. Normally, HSA is a simple protein without any sugar chains [21]. However, in the case of Albumin Casebrook, an *N*-glycan consensus sequence (Asn-Glu-Thr) appears due to the D494N mutation, resulting in an attachment of *N*-glycan chains at the asparagine position 494. The functions of Albumin Casebrook are not different from that of wild-type HAS [54]. The lifespan of families carrying Albumin Casebrook is also the same as those of wild-type HSA carriers [54]. Therefore, we speculate that albumin (D494N) rarely induces immunogenicity or mutagenicity.

The aim of this study was to evaluate the usefulness of type-I IFN-mannosylated albumin fusion protein. Although we have already produced human mannosylated albumin-type-I IFN, we created a mouse type-I IFN-mannosylated albumin fusion protein to improve the biological activity of type-I IFN (Figure 1). In fact, the induction ability of Man-MSA-mIFNα2 to induce IL-10, IL-1Ra, and PD-L1 in RAW264.7 cells was superior to those of the human fusion protein (Figure 2). This result is consistent with the reports where species differences between type-I IFN receptor on target cells and type-I IFN attenuated the biological activity derived from type-I IFN [30]. Furthermore, if a heterogeneous protein, e.g., hIFNα2b or HSA, is repeatedly administered to animal models with chronic disease, there is a concern that the drug’s efficacy may be diminished due to the appearance of antibodies against the foreign protein. In fact, chronic hepatitis model mice that repeatedly received Man-HSA-hIFNα2b showed large variations in ALT values (Supplemental Figure S1). Therefore, if an analysis of the biological actions of a protein formulation is performed based on repeat administrations, it is important to consider the species differences of the protein in the experimental animals.

Type-I IFN exerts various biological activities, such as antiviral, antitumor, and immunomodulatory effects [10], which are its core medicinal properties, through the type-I IFN receptor. In particular, the anti-inflammatory effects of type-I IFN have recently attracted attention in clinical practice [56]. Yoshida et al. found that patients who were treated with type-I IFN had a lower occurrence rate of hepatocellular carcinoma, and pointed out that the anti-inflammatory effect of type-I IFN may have contributed to this phenomenon. However, the anti-inflammatory effects of type-I IFN would be contradictory depending on the strength of signal transduction from type-I IFN receptors on immune cells [18]. Man-MSA-mIFNα2 did not induce inflammatory factors (Figure 5A), but induced anti-inflammatory factors (Figure 2), suggesting that the fusion protein accumulated at a high concentration on the type-I IFN receptor of macrophages as expected.

IL-1Ra is an endogenous anti-inflammatory substance that competes with IL-1β, an end product of the inflammasome system (a vital response of innate immunity), on the IL-1 receptor [57]. Research has revealed that inflammasomes are associated with various inflammatory diseases, such as liver disease [58], type 2 diabetes [59], amyloidosis [60], amyotrophic lateral sclerosis [61], and rheumatism [62], showing that they are a promising therapeutic target for chronic inflammatory diseases. Further, a recombinant IL-1Ra preparation has already been approved as a treatment for rheumatism [63] and several other drugs are also in clinical trials [64]. On the other hand, IL-10, a representative anti-inflammatory cytokine, suppresses the production of inflammatory cytokines and controls immune function by shifting the polarity of macrophages from the inflammatory type (M1) to the anti-inflammatory type (M2) [65,66]. As shown in Figure 5B, we isolated hepatic macrophages from CCl_4_-induced chronic hepatitis mice and analyzed the expression levels of CD80 (M1 markers) and CD206 (M2 markers) (Figure 5). The increased M1/M2 in the saline-administered group showed a reduction in the Man-MSA-mIFNα2-administered group (Table 1). These results imply that not only IL-10 but also IL-1Ra induced by Man-MSA-mIFNα2 cooperatively exerted an anti-inflammatory effect.

Although there are drugs that induce either IL-1Ra or IL-10, it is noteworthy that Man-MSA-mIFNα2 not only induces both, but also PD-L1 expression. As a result of the interaction of PD-L1 with PD-1 on T cells, PD-L1 induces immune tolerance and suppresses immune-related organ damage [67]. A previous study conducted by us revealed that Man-HSA-hIFNα2b improved hepatocellular damage with an increase in hepatic PD-L1 expression in concanavalin A-induced hepatitis model mice [31]. On the other hand, the pretreatment of these model mice with anti-PD-1 antibody exacerbated liver damage with an increase in plasma ALT values [31]. A similar phenomenon has been reported in clinical practice [68]. The administration of nivolumab, an anti-PD-1 antibody preparation, to squamous cell carcinoma patients, who were found to be positive for hepatitis C virus antibodies, caused an acute exacerbation of hepatitis C with an increase in plasma ALT values. Therefore, we speculate that PD-L1-induced immune tolerance also contributed to the hepatoprotective effect of Man-MSA-mIFNα2 in CCl_4_-induced chronic hepatitis.

Our previous studies have revealed that Man-HSA-hIFNα2b almost disappears from the blood 2 h after intravenous administration [31]. Since the half-life of Man-MSA-mIFNα2 is also expected to be several hours, repeated administration is required for Man-MSA-mIFNα2 to be effective in CCl_4_-induced chronic hepatitis model mice. Thus, using an explorative method, we administered Man-MSA-mIFNα2 to chronic hepatitis model mice twice a week (Figure 4A). In fact, administration of Man-MSA-mIFNα2 twice a week for 2 weeks to CCl_4_-induced chronic hepatitis model mice significantly reduced the serum ALT values (Figure 4B). Hepatic inflammation and liver fibrosis were also improved through repeated administrations of Man-MSA-mIFNα2 (Figure 5A). Therefore, therapeutic intervention with Man-MSA-mIFNα2 at the initial stage of chronic hepatitis would suppress the activation of Kupffer cells and HSCs, exerting hepatoprotective and antifibrotic effects. It should be noted that IL-1Ra, IL-10, and PD-L1 induced by type-I IFNs are likely to be involved in the therapeutic effects of Man-MSA-mIFNα2, but this point was not clarified in this study. In the future, the effects of combined treatment with Man-MSA-mIFNα2 and various inhibitors or neutralizing antibodies on CCl_4_-induced chronic liver injury model mice should be verified.

Although there are a lot of different pathogenic mechanisms among these patients with different etiologies, such as HBV or HCV, NASH or alcoholic liver disease, oxidative stress and inflammation play key roles in the onset and progression of liver diseases regardless of the etiology and natural course [69]. A common source of oxidative stress in liver diseases emanates from activated Kupffer cells, which contribute to the inflammatory responses in all forms of chronic liver diseases [70,71]. Activated Kupffer cells, through a nuclear factor κ-light-chain enhancer of activated B cells (NF-κB)-mediated mechanism, produce a complex and highly interactive repertoire of inflammatory mediators and cytokines [70], such as TNF-α, interleukins IL-1β, IL-6, IL-12, IL-18, and iNOS [72], as well as activate them to produce oxidants, including superoxide-derived nicotinamide adenine dinucleotide phosphate-oxidase (NADPH) and endocytose bacteria carried through the portal circulation [73]. Interestingly, under chronic liver diseases, endogenous type-I interferon acts on Kupffer cells, thus promoting the induction of anti-inflammatory cytokine or molecule including IL-10, IL-1Ra, and PD-L1 [13,14,74,75]. As evidence that type-I IFN-mannosylated albumin fusion protein suppressed inflammatory responses, our previous study revealed that Man-HSA-hIFNα2b suppressed reactive oxygen species (ROS) production in Kupffer cells [31]. Considering the findings in this study, coupled with the anti-inflammatory effect of type I IFN, type-I IFN-mannosylated albumin fusion protein is not a treatment to directly remove the causes, but is expected to exert therapeutic effects on liver diseases such as viral hepatitis, alcoholic hepatitis, and NASH. In fact, Tilg et al. reported that the inflammasome inhibitor IL-1Ra and anti-IL-1 antibody are effective against alcoholic hepatitis and NASH [76]. In the future, it will be necessary to verify the usefulness of Man-MSA-mIFNα2 in the pathological model of aforementioned liver diseases.

## 4. Materials and Methods

### 4.1. Materials

Plasmid purification kits (QIAGEN Plasmid Maxi Kit, QIAprep Spin Miniprep Kit) were purchased from QIAGEN (Venlo, The Netherlands). Restriction enzyme, T4 polynucleotide kinase, alkaline phosphatase (*E. coli* C75), DNA ligation kit (DNA Ligation Kit Ver.1), DNA polymerase (TAKARA Premix Taq, EX Taq version), and Site-Directed Mutagenesis kit (Mutan^®^- Super Express Km) were purchased from Takara Bio (Kusatsu, Japan). Heparin was purchased from Mochida Pharmaceuticals (Shinjuku City, Tokyo) and Block Ace from Sumitomo Dainippon Pharma (Osaka, Japan). Blue Sepharose 6-Fast Flow, 5 mL HiTrap Phenyl HP, and 5 mL HiTrap Q XL were purchased from GE Healthcare Japan (Tokyo, Japan). HE staining reagents were purchased from Muto Chemical (Tokyo, Japan). Mouse anti-α-SMA antibody (cat#:ab5694) was purchased from abcam (Cambridge, UK). All other reagents and solvents were commercially available special grade products, and the water used as the solvent was ion-exchanged water or Milli-Q water.

### 4.2. Animals

ICR mice (male, 4 weeks) were obtained from Japan SLC, Inc. (Shizuoka, Japan). Then, 10 mL/kg of CCl_4_ solutions (CCl_4_: corn oil  =  1:9) were administered intraperitoneally to the mice twice a week. Each fusion of type-I IFN with mannosylated albumin was administered at a dose of 300 nmol/kg twice a week from week 6 after starting the repeated administration of CCl_4_.

### 4.3. Cell Culture

RAW264.7 cells were cultured in DMEM medium containing 10% FBS, streptomycin, and penicillin and maintained at 37 °C and 5% CO_2_. The medium was changed at 3-day intervals. The cells were passaged with a cell scraper after reaching confluence.

### 4.4. DNA Recombination of MSA(D494N)-mIFNα2(N78Q) Fusion Protein

The designed fusion protein was composed of MSA(D494N) linked to mIFNα2(N78Q) via a polypeptide linker (-(GGGGS)_2_-). As previously reported, PCR was performed with a PfuTurbo DNA polymerase [31]. To isolate the DNA fragment of the base sequence cording for MSA, restriction enzyme Xho1 and Ava1 recognition regions were inserted into the 5′ terminal and the 3′ terminal, respectively. To isolate the DNA fragment of the base sequence coding for mIFNα2, restriction enzyme Ava1 and EcoR1 recognition regions were inserted into the 5′ terminal and the 3′ terminal, respectively. The pPIC9 was digested with Xho1 and EcoR1, and the appropriate side of the pPIC9 fragment was extracted by agarose gel electrophoresis. The cDNA construct cording for MSA-mIFNα2 was produced by ligating DNA fragments (pPIC9, MSA and mIFNα2) overnight at 16 °C. The mutation of MSA(D494N) and mIFNα2(N78Q) was performed using a Quick Change kit (Agilent, Santa Clara, CA, USA) with the mutagenic primers described in Table 2.

*Pichia pastoris* (SMD1168 strain) was transformed with Sal1-digested pPIC9-MSA(D494N)-mIFNα2(N78Q) by electroporation according to the manual [31].

### 4.5. Expression and Purification of the Fusion Protein

The expression and purification of Man-MSA(D494N)-mIFNα2(N78Q) was performed using the method previously established for fusion proteins [31].

### 4.6. Quantitative Real-Time Polymerase Chain Reaction (qRT-PCR)

Isolation of total RNA from RAW 264.7 cells or liver tissues and synthesis of cDNA were performed as previously described [31]. The gene expression levels for IL-10, IL-1Ra, PD-L1, TGF-β, Fibronectin, Snail, and Col1α2 were measured by qRT-PCR. All primers were purchased from Takara Bio (Tokyo, Japan); the sequences of the oligonucleotide primers are provided in Table 3.

### 4.7. Measurement of Alanine Aminotransferase (ALT)

ALT values, a biochemical marker of liver injury, in plasma were measured using a transaminase CII kit (Wako Chem., Saitama, Japan).

### 4.8. Measurement of Hepatic Hydroxyproline Contents

Livers were extracted from mice and homogenized in 1 mL of milli-Q water. After the supernatant was removed by centrifugation at 10,000 rpm at 4 °C for 5 min, the pellet was incubated with 500 μL of 10 N HCl at 110 °C for 16 h. The dried pellets were resuspended in 1 mL of milli-Q. An amount of 500 μL of the resulting suspension was incubated with a solution of chloramine-T (1.4% chloramine T, 4.1% sodium acetate, 10% Isopropanol) for 20 min at room temperature and Ehrlich’s reagent (1 M dimethyl benzaldehyde, 70% [*v*/*v*] isopropanol, and 30% [*v*/*v*] perchloric acid) for 15 min at 65 °C. The OD at 595 nm was measured using a microplate reader (Model 680; Bio-Rad Laboratories, Hercules, CA, USA).

### 4.9. Histological Analyses of Liver Tissue

For dehydration, 10% phosphate-buffered formalin-fixed liver tissues were processed with various concentrations of ethanol. Paraffin-embedded mouse liver tissue blocks were cut into 4 µm sections. The sections were subjected to hematoxylin and eosin (HE) and picrosirius red staining for morphologic analysis and the detection of collagen fibers, respectively. The sections were then observed using a microscope (BZ-8000; Keyence, Osaka, Japan).

### 4.10. Immunofluorescence Staining of Liver Tissue

The paraffin sections were treated with HistoVT One (Nacalai Tesque, Kyoto, Japan) at 95 °C for 30 min for antigen retrieval, then incubated with 4% Block Ace (KAC, Kyoto, Japan) at room temperature for 10 min. The sections were incubated with anti-α-SMA (1:100; abcam, Cambridge, UK) primary antibodies at 4 °C overnight. The primary antibody was visualized by secondary antibody (Alexafluor anti-rabbit 488, 1:200; Invitrogen, Carlsbad, CA, USA) followed by incubation for 90 min at room temperature. The sections were then observed using a microscope (BZ-8000; Keyence, Osaka, Japan).

### 4.11. Quantification of Hepatic TNF-α Levels

Livers were extracted from mice and homogenized in 1 mL of RIPA buffer (50 mM Tris/HCl (pH = 7.5), 5 M NaCl, 10% SDS, 10% Triton X-100, 10% sodium deoxycholate). TNF-α levels in the liver were measured via an enzyme-linked immunosorbent assay (EILSA) in accordance with the manufacturer’s recommended protocol (Biolegend, San Diego, CA, USA).

### 4.12. Analysis of M1/M2 Macrophage Polarization

Hepatic macrophages were isolated from the liver using previously described procedures [9,19]. To identify the M1 or M2 phenotypes, the hepatic macrophages were stained with antibodies against CD80 (1:50, PerCP-eFluor 710-conjugated anti-CD80 antibody; Thermo Fisher Scientific, Rockford, IL, USA) and CD206 (1:50, anti-CD206 antibody; R&D Systems, Minneapolis, MN, USA). The intensity of the fluorescent probe was determined using a Guava easyCyte flow cytometer (Merck Millipore, Burlington, MA, USA) with a 488 nm wavelength laser.

### 4.13. Statistical Analyses

Statistical analysis was undertaken using Prism 9 Software. The means for more than two groups were compared by one-way ANOVA followed by Tukey’s multiple comparison. A probability value of *p* < 0.05, *p* < 0.01 was considered to be significant. All data are represented as the average ± standard error.

## 5. Conclusions

The twice-weekly administration of Man-MSA-mIFNα2 was found to exhibit anti-inflammatory effects, inhibit the activation of hepatic macrophages and stellate cells, and improve the pathology of chronic liver damage. Therefore, type-I IFN-mannosylated albumin fusion protein has potential as a new therapeutic agent for various types of chronic hepatitis.

## Figures and Tables

**Table 1:** Effect of Man-MSA-mIFNα2 on M1/M2 polarization of hepatic macrophages in CCl_4_-induced chronic hepatitis mice.

Group	M1 (%)	M2 (%)	M1/M2
Control	27.06	29.82	0.90
Saline	77.59	3.01	25.78
Man-MSA-mIFNα2	66.76	8.71	7.66

**Table 2:** Sequence of mutagenic primers for point mutations of MSA(D494N) or mIFNα2(N78Q).

Mutation	Forward	Reverse
MSA(D494N)	ACATATGTTTCATTAACTGTCAGAG	CTCTGACAGTTAATGAAACATATGT
mIFNα2(N78Q)	AGGAGGGTTGCCTGCCAAGCAGCAG	CTGCTGCTTGGCAGGCAACCCTCCT

**Table 3:** Sequence of Primers for qRT-PCR.

Target Gene	Forward	Reverse
IL-10	GGACAACATACTGCTAACCGACTC	AAAATCACTCTTCACCTGCTCCAC
IL-1Ra	TCAGATCTGCACTCAATGCC	CTGGTGTTTGACCTGGGAGT
PD-L1	TCAGCTACGGTGGTGCGGACT	AGCTTCTGGATAACCCTCGGCCT
GAPDH	AACTTTGGCATTGTGGAAGG	ACACATTGGGGGTAGGAACA
TGF-β	GGATACCAACTATTGCTTCAGCTCC	AGGCTCCAAATATAGGGGCAGGGTC
Fibronectin	GGCCACACCTACAACCAGTA	TCGTCTCTGTCAGCTTGCAC
Snail	CAACTATAGCGAGCTGCAGGA	ACTTGGGGTACCAGGAGAGAGT
Col1α2	CACCCCAGCGAAGAACTCATA	GCCACCATTGATAGTCTCTCCTAAC
18S	GTAACCCGTTGAACCCCATT	CCATCCAATCGGTAGTAGCG

## Data Availability

Data is contained within the article and supplementary material.

## Supplementary Materials

The following supporting information can be downloaded at https://www.mdpi.com/article/10.3390/ph17020260/s1, Figure S1. Effect of each fusion of type-I IFN with mannosylated albumin on hepatocellular damage in CCl4-induced chronic hepatitis mice. (A) A schematic summary of the experimental protocol for evaluating the effect of each fusion protein on hepatocellular damage on CCl_4_- induced chronic hepatitis mice. (B) Plasma ALT values were determined 8 weeks after repeated administration of CCl4 (1.0 mL/kg, i.p.). Each value represents the mean ± S.E. (n = 5).
