# Supplementary material for: Mouse Type-I Interferon-Mannosylated Albumin Fusion Protein for the Treatment of Chronic Hepatitis
Source: Pharmaceuticals (Basel). 2024 Feb 19;17(2):260. doi: 10.3390/ph17020260 (PMC10893114; doi:10.3390/ph17020260)
Supplement: Supplementary file 1 [file pharmaceuticals-17-00260-s001.zip › pharmaceuticals-2828098-supplementary.pdf]

Supplemental Materials

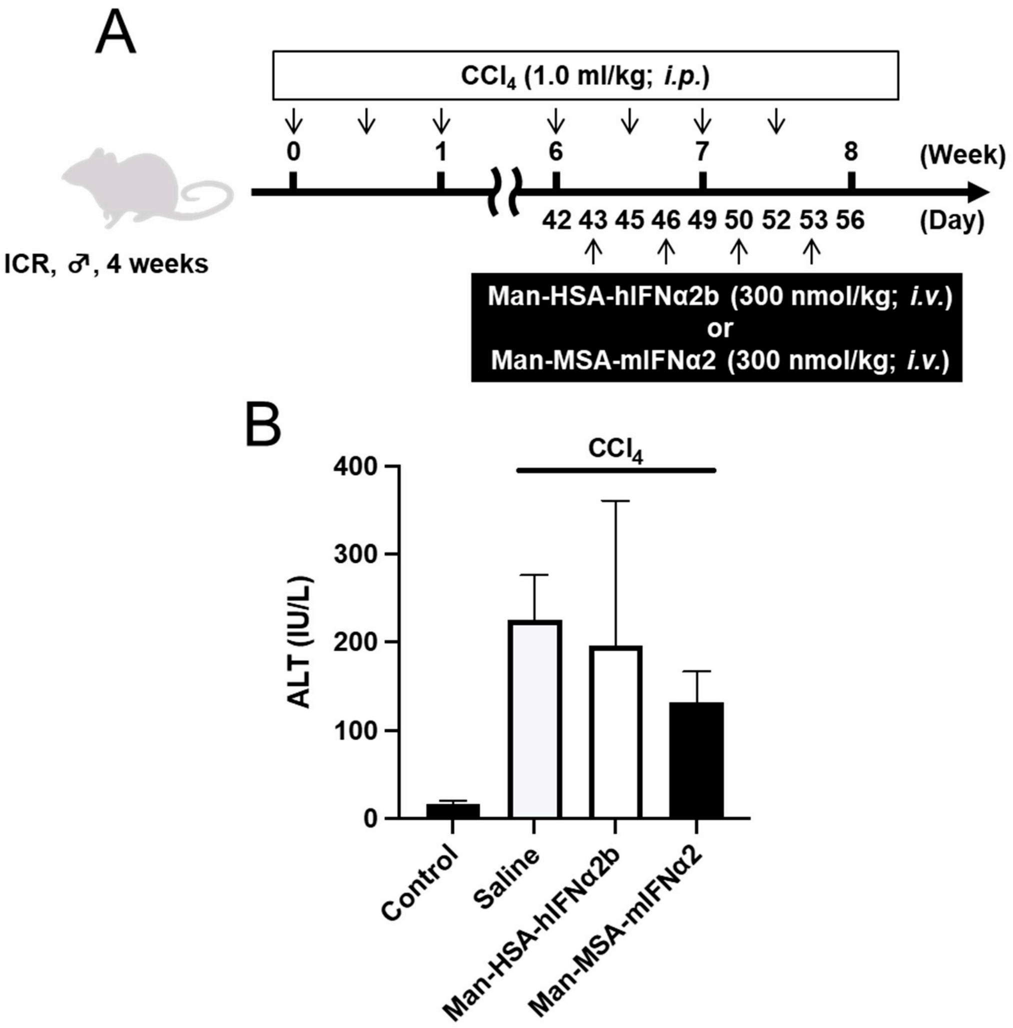

Figure S1. Effect of each fusion of type-I IFN with mannosylated albumin on hepatocellular damage in CCl_4-induced chronic hepatitis mice (A) Schematic summary of the experimental protocol for evaluation of the effect of each fusion protein on hepatocellular damage on CCl_4-induced chronic hepatitis mice. (B) Plasma ALT values were determined 8 weeks after repeated administration of CCl_4 (1.0 ml/kg, i.p.). Each value represents the mean ± S.E. (n=5).
